# Mean Changes in Estimated Glomerular Filtration Rate in Patients Undergoing Percutaneous Nephrolithotomy Having Renal Stone Disease

**DOI:** 10.7759/cureus.13328

**Published:** 2021-02-13

**Authors:** Sikandar Ali, Sajjad Ali, Umar Farooque, Saima Iqbal, FNU Farukhuddin, Rizwan Farooque, Kubiat Effiong, Kuseme Effiong, Muhammad Daim Bin Zafar, Mostafa A Shehata

**Affiliations:** 1 Urology, Sindh Institute of Urology and Transplantation, Karachi, PAK; 2 Neurology, Dow University of Health Sciences, Karachi, PAK; 3 Obstetrics and Gynaecology, Civil Hospital Karachi, Karachi, PAK; 4 Neurology, University Hospital Cleveland Medical Center, Cleveland, USA; 5 Internal Medicine, Sindh Medical College, Karachi, PAK; 6 Internal Medicine, V.N. Karazin National Medical University, Kharkiv, UKR; 7 Internal Medicine, Kharkiv National Medical University, Kharkiv, UKR; 8 Internal Medicine, Dow University of Health Sciences, Civil Hospital Karachi, Karachi, PAK; 9 Medicine and Surgery, Alexandria Faculty of Medicine, Alexandria, EGY

**Keywords:** estimated glomerular filtration rate, gfr, percutaneous nephrolithotomy, pcnl, renal stone disease

## Abstract

Background

Urinary stone disease is associated with renal impairment because of obstruction and infection. Comorbidities include hypertension, dyslipidemia, diabetes, and impaired renal function. Furthermore, as recurrences are common in urolithiasis, such patients undergo many treatments throughout their life. Percutaneous nephrolithotomy (PCNL) is an effective treatment for renal stones with a diameter greater than 2 cm. The primary objective of this study was to observe the mean changes in estimated glomerular filtration rate (GFR) in patients undergoing PCNL having renal stone disease.

Methodology

This cross-sectional study was conducted for six months between June and November 2020 at a tertiary care hospital in Karachi, Pakistan. All male and female patients aged between 15 and 70 years who were diagnosed with renal stones using X-ray of the kidney, ureter, and bladder or using ultrasound of the abdomen and planned for PCNL were selected. Patients with any duration of kidney stone disease were included. Statistical Package for Social Sciences version 20.0 (IBM Corp., Armonk, NY, USA) was used to statistically analyze the data.

Results

The mean age of the patients was 41.11 ± 14.30 (17-70) years. A total of 61 (38.1%) patients were female and 99 (61.9%) were male. Mean preoperative GFR was 91.22 ± 5.88 mL/min which decreased to 83.64 ± 5.70 mL/min at 48 hours post-PCNL. GFR significantly decreased after surgery (p = 0.0001).

Conclusions

During early postoperative days, GFR was decreased in patients undergoing PCNL. Factors that may impair renal function should be avoided during the first few days after undergoing PCNL. Further large-scale studies are needed to investigate these changes in GFR in post-PCNL patients.

## Introduction

Urolithiasis, also known as urinary stone disease, is always associated with some amount of renal impairment which can be due to infectious agents or obstructive agents in the urinary system. Hypertension, dyslipidemia, and diabetes have also been reported to affect renal function [1]. Frequent recurrences in renal stone patients lead to treatments throughout their lives. Renal stone disease and its comorbidities have been proven to affect renal parenchyma [2]. Therefore, the objectives of renal stone treatment should be to remove the stones and protect the kidneys as well.

Percutaneous nephrolithotomy (PCNL) is now regarded as an effective treatment for renal stones greater than 2 cm in diameter and is preferred over other procedures due to its less invasive nature as long as an appropriate route is chosen to prevent excessive bleeding [3]. Several studies have been conducted to determine the effects of PCNL on postoperative renal function. Varying results have been reported by studies regarding the effects of PCNL on postoperative renal function. Some of these studies reported PCNL treatment to have minimalistic effects on renal function in the early postoperative period [4-6]. Hosseini et al., on the other hand, found a remarkable decrease in glomerular filtration rate (GFR) after PCNL as the estimated GFR levels in their study decreased from 74.89 mL/min preoperatively to 64.04 mL/min 48 hours after PCNL treatment [7]. Nouralizadeh et al. also reported similar results as the estimated reduction in GFR within 48 hours after PCNL was from 87.5 ± 26.7 mL/min to 75.9 ± 25.0 mL/min [8]. Hence, this study was carried out to observe the effects of PCNL on estimated GFR within 48 hours after the principal procedure. This study will help healthcare professionals to determine whether any deleterious effects of PCNL exist on postoperative renal function and to design better treatment strategies to reduce mortality and morbidity in these patients.

## Materials and methods

Study design and setting

This cross-sectional study was conducted at a tertiary care hospital in Karachi, Pakistan, for six months between June and November 2020.

Sample size, inclusion, and exclusion criteria

The sample size was calculated to be 160 patients using Openepi at a confidence interval of 95%, statistical power of 80%, and effect size of 50% based on the results of a previously conducted study [7]. The sample size was increased to 160 patients to obtain a more representative sample from the general population. All patients aged 15-70 years of either gender, that is, males or females, diagnosed with renal stones by X-ray of the kidney, ureter, and bladder or by ultrasound of the abdomen and planned for PCNL were included in this study. Patients with any duration of kidney stone disease were included. All patients having stones associated with congenital anomalies of kidney and ureter diagnosed on ultrasound before surgery were excluded from the study.

Data collection and sampling technique

The non-probability consecutive sampling technique was used and all included patients signed informed consent. PCNL procedures were carried out by senior consultant urologists. PCNL was done using pneumatic lithotripsy which was done to fragment the stones into smaller pieces, which could be removed using a grasper. In all patients, preoperative estimated GFR and GFR after 48 hours of PCNL were measured. Data regarding confounding variables such as hypertension, diabetes, and dyslipidemia were also collected based on the previous history of patients. Data regarding age, gender, and duration of kidney stone disease were also obtained.

Statistical analysis

Statistical Package for Social Sciences version 20.0 (IBM Corp., Armonk, NY, USA) was used for data analysis. Mean and standard deviation were calculated for quantitative variables including age, preoperative GFR, postoperative GFR, and duration of renal stone disease. Categorical variables including gender, hypertension, diabetes, and dyslipidemia were presented using percentages and frequencies. Confounding variables included gender, age, duration of renal stone disease, hypertension, diabetes, and mean changes in estimated GFR. Any p-value of <0.05 was regarded significant.

## Results

Table 1 demonstrates the descriptive statistics of baseline characteristics of the patients enrolled in the study. Overall, 41.11 ± 14.30 (17-70) years was the mean age of the patients. However, the mean duration of renal stone disease was 2.5 ± 0.5 (1-4) months. A total of 61 (38.1%) patients were females and 99 (61.9%) were males. In total, 101 (63.1%) patients had renal stone symptoms for less than one month and 59 (36.9%) patients had a duration of greater than or equal to one month. A total of 48 (30%) patients were hypertensive, 55 (34.4%) were diabetic, and 36 (22.5%) had dyslipidemia.

**Table 1:** Descriptive statistics of baseline characteristics of patients. GFR: glomerular filtration rate; post-op: postoperative; pre-op: preoperative

Variables	n	Minimum	Maximum	Mean	Standard deviation
Age (years)	160	17	70	41.11	14.3
Duration of renal stone (months)	160	1	4	2.5	0.5
Pre-op GFR	160	79.2	102.8	91.22	5.83
Post-op GFR	160	70.5	98.9	83.64	5.7

As shown in Table 2, the mean preoperative GFR was 91.22 ± 5.88 mL/min which decreased to 83.64 ± 5.70 mL/min at 48 hours post-PCNL. GFR significantly decreased following surgery (p = 0.0001).

**Table 2:** Comparison of pre- and post-op estimated GFR. GFR: glomerular filtration rate; post-op: postoperative; pre-op: preoperative

Estimated GFR	n	Mean	Standard deviation	P-Value
Pre-op GFR	160	91.22	5.88	0.0001
Post-op GFR	160	83.64	5.70	

Table 3 shows the stratification of preoperative and postoperative GFR with respect to effect modifiers.

**Table 3:** Stratification of pre- and post-op GFR with respect to effect modifiers. GFR: glomerular filtration rate; post-op: postoperative; pre-op: preoperative

Variables			Mean	n	Standard deviation	P-Value
Age groups	<35 years	Pre-op GFR	90.76	60	6.09	0.0001
		Post-op GFR	83.34	60	5.68	
	≥35 years	Pre-op GFR	91.50	100	5.77	0.0001
		Post-op GFR	83.82	100	5.74	
Gender	Female	Pre-op GFR	90.32	61	5.80	0.0001
		Post-op GFR	83.21	61	5.37	
	Male	Pre-op GFR	91.78	99	5.88	0.0001
		Post-op GFR	83.90	99	5.91	
Duration of renal stone	<1 month	Pre-op GFR	91.20	101	5.73	0.0001
		Post-op GFR	82.88	101	5.48	
	≥1 month	Pre-op GFR	91.24	59	6.18	0.0001
		Post-op GFR	84.94	59	5.89	
Hypertension	No	Pre-op GFR	91.97	112	5.74	0.0001
		Post-op GFR	84.19	112	5.61	
	Yes	Pre-op GFR	89.46	48	5.88	0.0001
		Post-op GFR	82.35	48	5.75	
Diabetes mellitus	No	Pre-op GFR	90.69	105	6.12	0.0001
		Post-op GFR	83.12	105	5.86	
	Yes	Pre-op GFR	92.23	55	5.31	0.0001
		Post-op GFR	84.64	55	5.30	
Dyslipidemia	No	Pre-op GFR	91.36	124	5.84	0.0001
		Post-op GFR	83.84	124	5.82	
	Yes	Pre-op GFR	90.77	36	6.07	0.0001
		Post-op GFR	82.95	36	5.31	

## Discussion

PCNL is related to reduced morbidity but increased risk and postoperative complications. It is essential to take the postoperative damage of each modality on renal parenchyma and function into account when selecting PCNL or open surgery, especially in patients with a single functioning kidney.

Multiple studies have reported the impact of PCNL on mid- and long-term renal function. The scar formation following PCNL has been reported to be <1% of the renal parenchyma in animal studies [9]. Dawaba et al. conducted a cohort study on 65 children aged nine months to 16 years. No renal scarring in any patients was observed on technetium dimercaptosuccinic acid scan, while technetium diethylenetetraminepentaacetic acid scan showed a statistically significant increase in GFR [10]. In another study conducted by Nouralizadeh and colleagues, serum creatinine and GFR were evaluated every six, 24, 48, and 72 hours following PCNL [8]. Bayrak et al. reported the early post-PCNL GFR to increase from the mean value of 104.30 ± 37.30 mL/min preoperatively to the mean value of 112.38 ± 40.1 mL/min postoperatively [11]. In our study, the mean preoperative GFR was 91.22 ± 5.88 mL/min which decreased to 83.64 ± 5.70 4mL/min at 48 hours post-PCNL. Hence, our results further reinforce the fact that GFR decreases during the first day (and the second day) after PCNL as we found a slight decrease in the very early hours after tubeless PCNL (<48 h), which could be attributed to the minimal renal parenchymal damages due to kidney dilation made to access the pyelocaliceal system during PCNL procedure [8,11].

It is recommended to avoid the use of nephrotoxic agents during the early postoperative period, particularly in patients with a single kidney, and those with comorbidities such as diabetes mellitus. Additionally, volume deficits should be adequately replaced to support susceptible kidneys.

Our study has a few limitations. Evaluation of preoperative GFR and differential renal function using renal scans were not possible due to ethical issues. Second, our method of occluding the ureter may seem unreliable but we tried to overcome this problem by confirming ureteral occlusion using antegrade nephrostography under fluoroscope. Finally, due to the relatively small number of patients, our study is a preliminary one and more sophisticated results would be achieved by conducting larger trials.

Further investigation might be valuable to evaluate changes in neurotransmitter levels; the effect of demographic data, position type, and anesthesia technique on GFR; and the role of various vasodilators in offsetting potential hemodynamic changes following PCNL.

## Conclusions

PCNL decreases the GFR during the early phase of the postoperative period. Factors that may impose a negative impact on renal function should be avoided. Factors such as age, hypertension, diabetes, dyslipidemia, duration of the stone, and nephrotoxic agents can affect renal function, and a study should be conducted with a larger sample size to extrapolate these results.

## References

[1] Shoag J, Halpern J, Goldfarb DS, Eisner BH (2014). Risk of chronic and end stage kidney disease in patients with nephrolithiasis. J Urol.

[2] Bos D, Abara E, Parmar MS (2014). Knowledge, attitudes, and practice patterns among healthcare providers in the prevention of recurrent kidney stones in Northern Ontario. Can Urol Assoc J.

[3] Ullah S, Ali S, Karimi S (2020). Frequency of blood transfusion in percutaneous nephrolithotomy. Cureus.

[4] Lechevallier E, Siles S, Ortega JC, Coulange C (1993). Comparison by SPECT of renal scars after extracorporeal shock wave lithotripsy and percutaneous nephrolithotomy. J Endourol.

[5] Samad L, Qureshi S, Zaidi Z (2007). Does percutaneous nephrolithotomy in children cause significant renal scarring?. J Pediatr Urol.

[6] Pérez-Fentes D, Cortés J, Gude F, García C, Ruibal Á, Aguiar P (2014). Does percutaneous nephrolithotomy and its outcomes have an impact on renal function? Quantitative analysis using SPECT-CT DSMA. Urolithiasis.

[7] Hosseini SR, Mohseni MG, Roshan H, Alizadeh F (2015). Effect of tubeless percutaneous nephrolithotomy on early renal function: does it deteriorate?. Adv Biomed Res.

[8] Nouralizadeh A, Sichani MM, Kashi AH (2011). Impacts of percutaneous nephrolithotomy on the estimated glomerular filtration rate during the first few days after surgery. Urol Res.

[9] Traxer O, Smith TG, Pearle MS, Corwin TS, Saboorian H, Cadeddu JA (2001). Renal parenchymal injury after standard and mini percutaneous nephrostolithotomy. J Urol.

[10] Dawaba MS, Shokeir AA, Hafez AT (2004). Percutaneous nephrolithotomy in children: early and late anatomical and functional results. J Urol.

[11] Bayrak O, Seckiner I, Erturhan SM, Mizrak S, Erbagci A (2012). Analysis of changes in the glomerular filtration rate as measured by the cockroft-gault formula in the early period after percutaneous nephrolithotomy. Korean J Urol.

